# Baseline Hepatic Levels of miR-29b and Claudin are Respectively Associated with the Stage of Fibrosis and HCV RNA in Hepatitis C

**Published:** 2019-03-08

**Authors:** Hossein Sendi, Marjan Mehrab-Mohseni, Mark W. Russo, Nury Steuerwald, Carl Jacobs, Mark G. Clemens, Herbert L. Bonkovsky

**Affiliations:** 1 The Liver-Biliary-Pancreatic Center, Carolinas Medical Center, Charlotte, NC, USA; 2 Department of Biology, University of North Carolina at Charlotte, Charlotte, NC, USA; 3 Department of pathology, Carolinas Medical Center, Charlotte, NC, USA; 4 Center for Nanotechnology in Drug Delivery, UNC School of Pharmacy, Chapel Hill, NC, USA; 5 Division of Gastroenterology, Department of Internal Medicine, Wake Forest University Medical Center, Winston-Salem, NC, USA

**Keywords:** micro RNA, miRNA, miR-122, HCV, Occludin, HCV RNA

## Abstract

We sought to determine if the baseline hepatic levels of miR-122, miR-29b, Claudin, Occludin, Protein Kinase R (PKR) or PKR activator (PRKRA) were correlated with HCV RNA or stage of fibrosis in patients with chronic hepatitis C (CHC). A total of 25 CHC patients (genotype 1) who were treatment naive at the time of sample collection enrolled in this study. By multivariate analysis, CLDN RNA was found as the single independent factor positively correlated with HCV RNA levels (p=0.003), while hepatic miR-29b levels was found as the single independent factor for predicting advanced stage of fibrosis (p=0.028). Conclusion: Our results highlight miR-29b and CLDN as novel predictors of advanced stage of liver fibrosis and baseline HCV RNA in CHC.

## Introduction

Various studies on the relationship between Hepatitis C Virus (HCV) biology and host miRNAs have demonstrated that HCV itself takes advantage of some miRNAs for its replication. The role of miR-122 in supporting HCV replication has already led to the development of an experimental anti-HCV drug [1]. Also, growing evidence shows several miRNAs play an important role in outcome of HCV infection [2]. The family of miRNA-29 plays role in modulating severity of liver disease. Recently, it was reported that treatment of hepatic stellate cells with TGFβ suppressed miR-29 expression suggesting that part of the fibrogenic effects of TGFβ is mediated via miR-29 down-regulation [3]. It was also shown that fibrosis and mortality were enhanced in miR-29 knockout mice in response to carbon tetrachloride [4]. In another recent study, it was shown that HCV infection down-regulates miR-29 in hepatocytes and may potentiate collagen synthesis by reducing miR-29 levels in activated HSCs [5].

Besides the regulatory role of miR-29 in fibrosis and cancer progression, the miR-29 family has been identified to have significant effect on immune cell proliferation and cytokine production by helper T cells, especially through targeting IFN- γ [6]. We sought other potential target genes for miR-29b and found that Claudin (CLDN), the tight junction component, an important player in the HCV entry into hepatocytes is among candidate targets of miR-29. On the other hand, miR-122, the most abundant hepatic miRNA, increases the abundance of HCV RNA [7]. We recently found that miR-122 binds to the 3’ UTR of OCLN, the other major HCV entry receptor, and decrease HCV entry into hepatocytes [8]. PKR, a double-stranded RNA-dependent protein kinase, is among the well-known members of cellular antiviral proteins transcriptionally induced by IFNs [9].

In a recent study, PKR activator (PRKRA) has been found to be targeted by miR-122 [10]. Based on our findings and previous studies, we studied levels of hepatic miR-122, miR-29b as well as hepatic mRNA levels of CLDN, OCLN, PKR, and PRKRA, to determine if there are any associations among pre-treatment levels of any of these molecular factors with HCV RNA levels, response to treatment and/or stage of liver disease.

## Patients and Methods

### Patients

The study included 25 patients with CHC (HCV genotype 1) followed in the Liver-Biliary-Pancreatic Center of Carolinas Medical Center (CMC) who agreed to participate in this study between 2008 and 2012. To be eligible for this study, patients had not been treated before study, and had been considered candidates for treatment after liver biopsies had been performed. Patients with autoimmune hepatitis, alcohol-induced liver injury, or hepatitis B virus-associated antigen/antibody or anti-human immunodeficiency virus antibody were excluded. Patients with a history of prior antiviral treatment for HCV before enrolment in the study were excluded.

Baseline levels of HCV RNA in serum were quantified and baseline liver biopsy specimens (obtained for routine clinical evaluation and care) were collected from patients not later than one week prior to starting treatment. Histological grading and staging of liver biopsy specimens from the CHC patients were provided by expert hepato-pathologists at CMC who were blinded as to other clinical or laboratory features. Baseline blood tests were conducted to determine each patient’s level of liver function test, and liver chemistries.

### Laboratory Methods

#### RNA isolation and miRNA RT-PCR from liver biopsy samples

Total RNA was isolated from liver biopsy samples using Trizol Reagent from Invitrogen Corp. (Carlsbad, CA, USA) per manufacturer’s instructions. The integrity of the RNA was verified by an Agilent 2100 Bioanalyzer profile from Agilent Technologies Inc. (Santa Clara, CA, USA).

For miRNA, first-strand complementary DNA synthesis was performed using TaqMan^®^ MicroRNA Reverse Transcription Kit primed with miR-specific primers from Applied Biosystems (Grand Island, NY, USA). Real-time quantitative RT-PCR (qRT-PCR) was performed using TaqMan^®^ MicroRNA Assays (Applied Biosystems), following the manufacturer’s recommendations with an ABI Prism 7500 Sequence Detection System using TaqMan^®^ Universal Master Mix (Applied Biosystems). Fold change values were calculated by comparative Ct analysis and normalized to SNORD44 concentrations. SNORD44 was used as an invariant control.

#### RT-PCR

qRT-PCR was performed as previously described [11]. GAPDH primers were designed as described [11]. The following primers were used for OCLN, and PRKRA. OCLN: forward primer 5’CTCCCGTTTGGATAAAGA3’; OCLN Reverse primer 5’TGATGTGTGACAATTTGCTC3’; PRKRA forward primer 5’AAGAAGCTGGCGAAACATAG3’; PRKRA reverse primer 5’GCCAATTCCTGTAATGAACC3’.

### Statistical Methods

For data measured on the interval scale (e.g. mir-29 levels), the Student’s t-test was used for comparing the means of 2 groups (e.g. gender). Analysis of variance, followed by Tukey’s test when appropriate, was used when comparing more than 2 groups. For the data with ordinal or not normally distribution the Wilcoxon rank sum test or the Kruskal-Wallis test was employed.

For nominal data, the chi-square or Fisher’s exact test was employed. To test for linear relationships among the variables measured on the interval (ordinal) scale, Pearson’s (Spearman’s) correlation coefficients were calculated. Multivariate analyses will include analysis of covariance for interval data, logistic regression for dichotomous outcomes (fibrosis stage 3-4 vs. stage 0-2) or general linear model (GLM) SPSS version 21 was used for all analyses. A two-tailed p-value of less than 0.05 was considered statistically significant.

## Results

Demographic, biochemical and virological data of the patients are shown in Table S1 A total of 25 patients with CHC were included in this study which were all treatment naive at the time of sample collection.

Then, we compared selected molecular factors in CHC patients with different stages of hepatic fibrosis. For this, we divided CHC patients into two categories; those with Metavir stages 0-2 of fibrosis on their pre-treatment biopsies and those with Metavir stages of 3-4 on their baseline liver biopsies. There was not a significant difference in age, sex or race between these two groups. The levels of hepatic miR-122 were slightly higher in patients with lower stages of fibrosis (55 *vs*. 50, p>0.05) which was not statistically significant. However, we found that CHC patients with higher stages of fibrosis had strong trend of higher hepatic miR-29b levels than patients with lower stages of fibrosis (0.215 *vs*. 0.120, p=0.057).

A multi-variable logistic regression analysis was done with age, sex, race, and hepatic levels of miR-122, and miR-29b considered as different factors and covariates while high stage of fibrosis (Stage 3-4) was considered as a single dependent variable. This multivariate analysis showed the hepatic miR-29b levels as the only independent factor for predicting advanced stage of fibrosis (p=0.028, Table 1).

We also investigated whether there is any correlation between hepatic levels of miR-122, or miR-29b with baseline HCV RNA levels.

**Figure 1:**
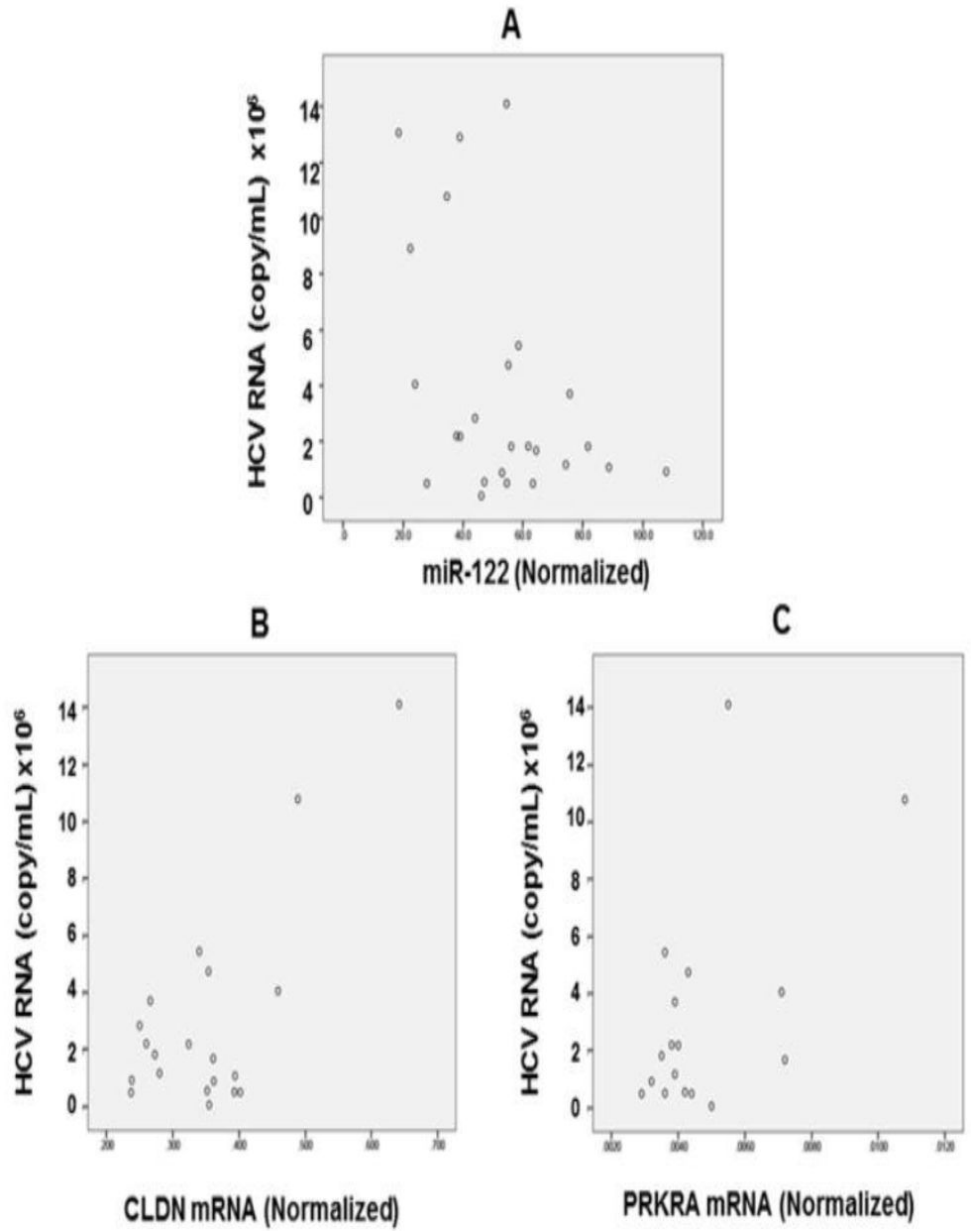
Levels of HCV RNA at baseline are negatively correlated with hepatic miR-122 levels while positively correlated with both CLDN, and PRKRA RNA levels. **Note:** Scatter plots correlate HCV RNA levels on Y axis with hepatic miR-122 levels (Figure A), CLDN mRNA (Figure B), or PRKRA RNA (Figure C) on X axis. Pearson correlation coefficients show significant inverse correlation of HCV RNA with miR-122 (r=−0.43, p=0.03; Figure A), but direct correlation with CLDN RNA (r=0.71, p<0.001; Figure B), and PRKRA RNA (r=0.55, p=0.02; Figure C) with HCV RNA levels respectively.

There was no correlation between hepatic levels of miR- 29b and HCV RNA levels, while we found that hepatic levels of miR-122 is negatively correlated with the baseline HCV RNA levels (r=−0.43, p=0.03; Figure 1). Interestingly, we found that hepatic levels of CLDN RNA are positively and strongly correlated with HCV RNA before starting the treatment (r=0.71, p<0.001; Figure 1). There was also a positive correlation between hepatic levels of PRKRA RNA and HCV RNA levels (r=0.55, p=0.02; Figure 1). We constructed a general linear model using regression analysis and analysis of variance for the baseline HCV RNA levels as single dependent variable (Table 2).

Age and sex were considered as factors and miR-122, CLDN RNA, PRKRA RNA and stage of fibrosis as covariates. The whole model was statistically significant (p<0.01). Hepatic CLDN RNA was found as the single independent factor which positively correlated with HCV RNA levels (F=15.7, p=0.003) while hepatic miR-122 levels showed a strong trend or borderline negative correlation with HCV RNA levels (F=4.8, p=0.056).

## Discussion

Investigating different molecular factors in 25 patients with CHC, we found hepatic CLDN RNA as the single independent factor, which positively correlated with HCV RNA levels in CHC patients. Besides, we found that hepatic levels of miR-29b may serve as an independent predictor of advanced stage of liver disease in patients with CHC.

The family of miRNA-29 plays a role in modulating severity of liver disease. It was also shown that fibrosis and mortality were enhanced in miR29 knockout mice in response to carbon tetrachloride [4]. In another recent study, it was shown that HCV infection down-regulates miR-29 in hepatocytes and may potentiate collagen synthesis by reducing miR-29 levels in activated HSCs [5]. We showed that miR-29b level serves as an independent factor for predicting advanced stage of fibrosis. These findings are unexpected, because, as just described, miR-29b has been shown to exhibit anti-fibrotic effects in vitro. Hence, caution should be exercised in extrapolating in vitro observations to subjects with CHC. Nevertheless, these findings highlight the importance of miR-29b as a novel predictor of advanced stages of liver fibrosis in patients with CHC. Higher baseline levels of hepatic mir-29b in patients with advanced liver fibrosis may indicate two theories. The first theory is that these patients might already had the maximum expression of hepatic miR-29b even before HCV infection, so further increase in expression of miR-29b during the course of infection could not be achieved as patients with lower baseline expression of hepatic miR-29b. The second theory is that these patients need to over-express miR-29b during course of infection because of severity of the liver disease but they could not avoid advanced liver fibrosis due to some other molecular factors. We understand that we analyzed a limited number of patients (25 patients) with CHC, but we are expecting similar future studies with more CHC patients would confirm the result of our current study.

## Conclusion

In summary, we found that baseline levels of hepatic miR-29b are associated with advanced stage of fibrosis in CHC patients. We also found CLDN RNA is positively associated with HCV RNA at baseline. Taken together, our results highlight hepatic miR-29b and CLDN as novel predictors of advanced stage of liver fibrosis and baseline HCV RNA in CHC.

## Supplementary Material

1

## Figures and Tables

**Table 1:** Among several factors studied only miR-29b was surprisingly predictive of hepatic fibrosis.

Variable	Univariate p-value	Multivariate p-value
Age	0.487	0.701
Sex	0.116	0.102
Race	0.107	0.275
miR-122 levels in liver	0.644	0.286
miR-29b levels in liver	0.057	0.028*

**Note:** Univariate and multivariate analysis for advanced stage of fibrosis (Stage 3-4) as dependent variable. Hepatic micro RNA levels were measured before the start of treatment (at week 0);

* (p<0.05).

**Table 2:** General linear model (GLM) analysis for HCV RNA as dependent variable.

Variable	CC	p-value	GLM F-value	GLM p-value
Age	0.05	0.82	0.02	0.882
Sex	0.11	0.613	0.11	0.753
Fibrosis stage	0.04	0.86	2.82	0.127
miR-122 level	−0.47	0.030*	4.8	0.056
CLDN RNA	0.71	0.001**	15.71	0.003**
PRKRA RNA	0.55	0.020*	0.53	0.458

**Note:** All biochemical, molecular, and viral data measured before the start of treatment (at week 0) were included in the model. GLM: General Linear Model, CC: Correlation Coefficient

* represents significant (p<0.05)

** represents significant (p<0.01).
