# Supplementary material for: Baseline Hepatic Levels of miR-29b and Claudin are Respectively Associated with the Stage of Fibrosis and HCV RNA in Hepatitis C
Source: Clin Exp Gastroenterol Hepatol. Author manuscript; Available in PMC 2019 Apr 4. (PMC6448799)
Supplement: 1 [file NIHMS1018699-supplement-1.pdf]

Supplementary Table 1: Demographic, biochemical data and HCV RNA levels at baseline in all 25 patients with CHC in the study.

Patient Characteristics	
Total (n)	25
Age (y)	53.3 ± 1.3
Male; Female	18;7
Caucasian; AA	16;9
ALT(IU/L)	76.1±15.1
AST(IU/L)	59 ± 8.4
HCV RNA [IU/mL]	3,929,000 ± 879000
Note: *AA indicates African-American, results are mean ± SEM.*	
